# Anti-apoptotic effect of HCV core gene of genotype 3a in Huh-7 cell line

**DOI:** 10.1186/1743-422X-8-522

**Published:** 2011-11-23

**Authors:** Shah Jahan, Saba Khaliq, Muhammad Hassan Siddiqi, Bushra Ijaz, Waqar Ahmad, Usman A Ashfaq, Sajida Hassan

**Affiliations:** 1 Centre of Excellence in Molecular Biology, University of the Punjab, Pakistan; 2 Department of Immunology, University of Health Sciences Lahore, Pakistan

## Abstract

**Background:**

Hepatitis C virus (HCV) Core protein regulates multiple signaling pathways and alters cellular genes expression responsible for HCV induced pathogenesis leading to hepatocellular carcinoma (HCC). Prevalence of HCV genotype 3a associated HCC is higher in Pakistan as compare to the rest of world; however the molecular mechanism behind this is still unclear. This study has been designed to evaluate the effect of HCV core 3a on apoptosis and cell proliferation which are involved in HCC

**Methodology:**

We examined the in vitro effect of HCV Core protein of genotype 3a and 1a on cellular genes involved in apoptosis by Real time PCR in liver cell line (Huh-7). We analyzed the effect of HCV core of genotype 1a and 3a on cell proliferation by MTT assay and on phosphrylation of Akt by western blotting in Huh-7 cells.

**Results:**

The HCV 3a Core down regulates the gene expression of Caspases (3, 8, 9 and 10), Cyto C and p53 which are involved in apoptosis. Moreover, HCV 3a Core gene showed stronger effect in regulating protein level of p-Akt as compared to HCV 1a Core accompanied by enhanced cell proliferation in Huh-7 cell line.

**Conclusion:**

From the current study it has been concluded that reduced expression of cellular genes involved in apoptosis, increased p-Akt (cell survival gene) and enhanced cell proliferation in response to HCV 3a core confirms anti apoptotic effect of HCV 3a Core gene in Huh-7 that may lead to HCC.

## Introduction

Hepatitis C virus (HCV) causes acute and chronic hepatitis which can lead to Hepatocellular carcinoma (HCC) in a significant number of patients via induction of oxidative stress, steatosis, insulin resistance, fibrosis and liver cirrhosis [1,2]. HCV is a major health problem with almost 350 million chronically infected individuals worldwide [3], and 10% of the Pakistani population being chronically infected with this viral pathogen with a higher prevalence rate of HCV association HCC as compared to the rest of the world [4,5]. HCV has a positive single-stranded RNA genome of approximately 9.5 kb that encodes a large precursor polyprotein of approximately 3000 amino acids. Among the processed HCV proteins, the Core protein of 191 amino acids is essential for nucleo-capsid formation in the viral particle [6]. HCV Core protein exerts many biological functions in the host cell, such as cellular growth, malignant transformation and apoptosis, which may be involved in HCV-related liver diseases by modulation of gene transcription, cell proliferation, and cell death leading to oxidative stress, liver steatosis and eventually HCC [7-9]. More interestingly, constitutive expression of HCV Core protein induced HCC in transgenic mice; the expression level of HCV Core protein in the liver of these mice was similar to that in patients with chronic hepatitis C [10].

Apoptosis is central for the control and elimination of viral infections. There are increasing evidences suggesting that liver cell damage in chronic HCV infection is mediated by apoptosis [11]. Enhanced apoptosis of hepatocytes has been described during chronic HCV infection and correlates with the degree of liver damage [12,13]. HCV induced apoptosis is mediated by both external and internal pathways. The impact of apoptosis in chronic HCV infection is not well understood. It may be harmful by triggering liver fibrosis, or essential in interferon (IFN) induced HCV elimination. For virtually all HCV proteins, pro- and anti-apoptotic effects have been described, especially for the Core and E2 protein [13]. HCV Core has pro- and anti-apoptotic effects in death ligand e.g., TNF-α and CD95 ligand mediated hepatocyte apoptosis has been described in a hepatoma cell line [14,15]. In contrast, HCV Core protein inhibits CD95 Ligand-mediated apoptosis by preventing Cytochrome C (Cyto C) release from mitochondria and consecutive activation of Caspase-9, 3 and 7 [16]. Several studies demonstrated binding of the HCV Core protein to p53, either inhibiting or activating p53 following with anti- or pro-apoptotic effects [17,18]. HCV counteracts p53 growth suppression through activation of MAPK and PI3K/Akt signaling cascades which enhances cell growth and proliferation [19]. In some studies apoptosis was inhibited in hepatoma through Core-dependent phosphorylation and activation of STAT3 that induces the anti-apoptotic bcl-XL [20].

Caspase-3, 8, 9 and 10, (proteases involved in apoptosis signaling cascade), Cyto C and p53 interaction is important in apoptosis [11]. Very few studies have been undertaken with respect to the HCV different genotypes and further studies are needed in this connection so as to fully expose the ambiguity of different response in different genotypes. In mammals, regulation of caspase in cell death is complex, however, Caspase-2, 8, 9 and 10 are initiator caspase, whereas Caspase-3, 6 and 7, serve as effecter caspases and Caspase-8 as the key initiator of death receptor-mediated apoptosis [21-23]. Cyto C is associated with Caspase-9 in intrinsic apoptosis pathway while p53 tumor suppressor gene is an important marker in apoptosis [18,24]. Expression of caspase family is higher in HCV infection, and enhanced hepatocyte apoptosis occurs through the intrinsic apoptosis pathway via mitochondria [25,26]. Studies of caspases were better alternative for investigating apoptosis in HCV infection as caspase activation occurs earlier than DNA cleavage [21,22].

Previously, we have shown the increased cell proliferation in HCV 3a Core transfected liver cells to understand the molecular mechanisms underlying the multifunction of HCV Core protein, and HCV 3a Core protein activates Cox-2 and VEGF more as compared to genotype 1a [27]. Akt acts as an important signal mediator, which regulates cell survival and proliferation [28,29]. The mechanisms of cell proliferation and transformation are intrinsically linked to the process of apoptosis. The Ras/phosphatidylinositol-3-kinase (PI3K)/Akt pathway is critical in anti-apoptotic signaling [30]. In this pathway, the serine-threonine kinase Akt directly phosphorylates many substrates, including Bad, Caspase-9, CREB, Forkhead and GSK-3, and is critical for cell survival in response to growth factor stimulation.

In general, apoptosis is central to viral clearance. In HCV-infected liver, however, despite enhanced hepatocyte apoptosis, viral persistence is observed with different frequencies in different genotypes, as due to the lack of proofreading function of the RNA-dependent RNA-polymerase (NS5B), HCV has a high mutation rate and exists as genetically heterogeneous quasispecies in individual patients [31-33]. To examine the role of HCV Core protein on caspase signaling, we investigated the expression levels of molecules downstream caspase signaling including Caspases-3, 8, 9, and 10, Cyto C and p53 which are most important in apoptotic pathway in transfected Huh-7 cell line. Moreover, we examined the activation of the PI(3)K/Akt pathway in cells expressing HCV Core 1a and 3a protein.

## Results

### Effect of HCV Core gene on gene expression of Caspases, Cyto C and p53

**Figure 1:**
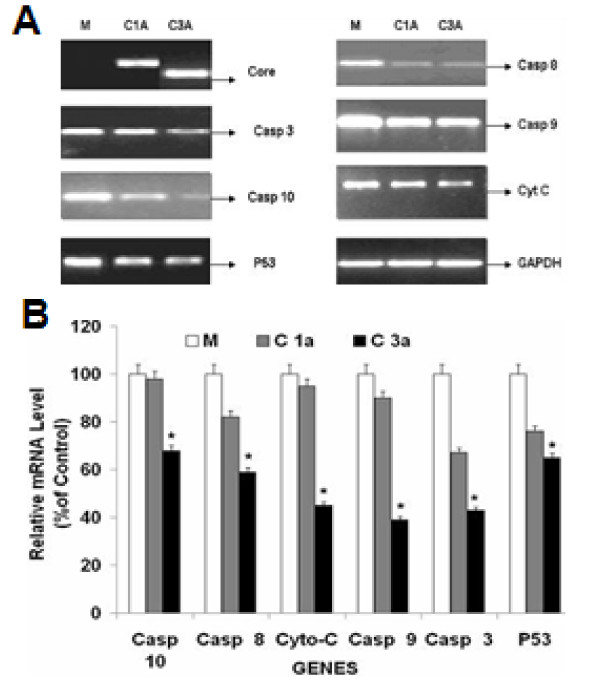
**Effect of HCV Core on the gene Expression of Caspase-3, 8, 9 and 10, Cyto C and p53 genes in Huh-7**. **A) **Huh-7 cells transiently transfected with HCV Core 1a (C1a), Core 3a (C3a) vectors and pCR3.1 plasmid alone as mock (M) samples (0.4 μg DNA/well of each plasmid). Cells were harvested after 48 hr and relative RNA determinations were carried out using semi-quantitative RT-PCR. **B) **Comparison of gene expression Caspase-3, 8, 9, 10, Cyto C and p53 genes of in transiently transfected Huh-7 cells. All experiments were performed in 3 independent experiments having triplicate samples in each. Error bars indicate, mean S.D, *p < 0.01 verses mock.

Monitoring of caspase activation might provide a reliable diagnostic tool to detect the degree of HCV-mediated inflammatory liver damage and to evaluate the effect of HCV genes on apoptotic pathways. The mRNA expression of Caspase-3, 8, 9 and 10, Cyto C and p53 genes in Core (genotype 1a and 3a) transfected Huh-7 cells was observed. Results indicated a reduced level of expression for these genes in genotype 3a Core (Core 3a) transfected cells as compared to the control and genotype 1a Core transfected cells (Figure 1A). Further, we analyzed sequences of HCV Core 1a and 3a protein clones which we used in these experiment with consensus reported sequence we found a significant nucleotide difference in sequences of HCV core 1a and 3a. (Additional File 1) These Expression of HCV core 1a and 3a were more profound in results obtained from Semi quantitative method and Real-Time PCR at 48 hr as compare to 72 and 96 hr (Additional File 2). Anti apoptotic effect of HCV core 3a on cellular genes mainly involved in apoptosis were more reflective in results obtained from Real-Time PCR at 48 hr (Additional File 3). The effect of HCV Core gene of genotype 1a and 3a on expression of cellular genes Caspase-3, 8, 9 and 10, Cyto C and p53 were detected by Real-Time PCR using gene specific-primers and SYBR Green mix (Fermentas). A more reduced expression of Caspase-3, 8, 9 and 10, Cyto C and p53 genes was observed in Core-transfected cells for genotype 3a as compared to genotype 1a. Reduction in the expression of Caspase-3, 8, 9 and 10, Cyto C and p53 genes were observed i.e., 60%, 40%, 60%, 35%, 60%, 30% respectively as compare to mock transfected cells (Figure 1B).

### HCV 3a Core gene decreases expression of Caspase-3 at protein

**Figure 2:**
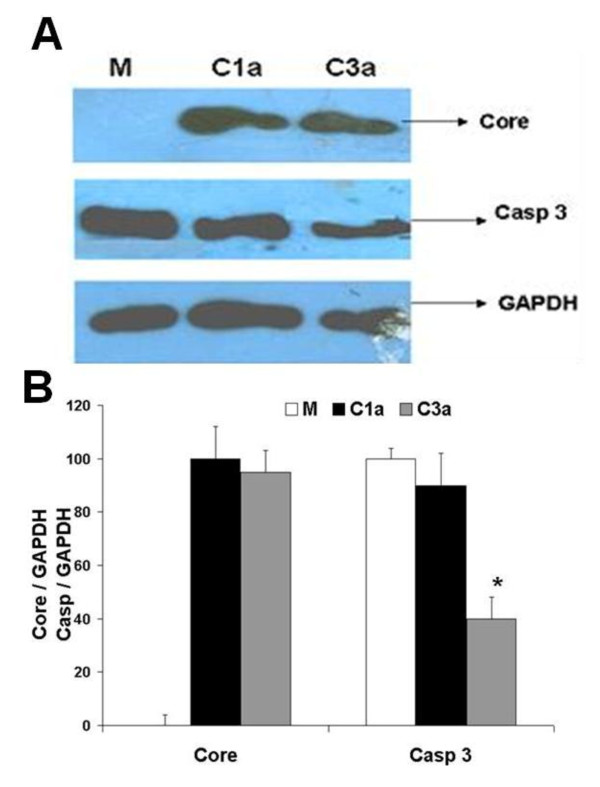
**HCV 3a Core gene decreases expression of Caspase-3 at protein level**. **A) **Protein expression levels were determined by western blot analysis from Huh-7 cell lysates after 48 hr transfection with HCV Core 1a (C1a) and Core 3a (C3a) using specific antibody. Protein levels for GAPDH gene are shown as internal control. **B) **Blots were also normalized by measuring the amount of GAPDH (histogram). Bars are mean of optical density ratio of each group.* p < 0.001 verses control and Core 1a. GAPDH gene was used as internal control for normalization in western blotting. All experiments were performed in 3 independent experiments having triplicate samples in each.

Caspase-3 is the most important down stream effecter triggered by Caspase-8, a vital component of the extrinsic cell death pathway, so we determined the protein expression levels of Caspase-3 gene for both genotypes (HCV 1a and 3a). Cell lysates from Huh-7 cells, transfected with HCV 1a and 3a Core plasmid were examined by western blot analysis using Core, Caspase-3 and GAPDH specific antibodies. There was a significant reduction in the expression levels of Caspase-3 protein in HCV 3a Core transfected Huh-7 cells as compared to HCV 1a (Figure 2A). Furthermore, by using densitometer analysis we confirmed that there was 55% reduction in protein level in Huh-7 cells transfected with Core 3a as compare to mock (M) (Figure 2B).

### HCV 3a Core protein activates the pAkt and increases cell proliferation

**Figure 3:**
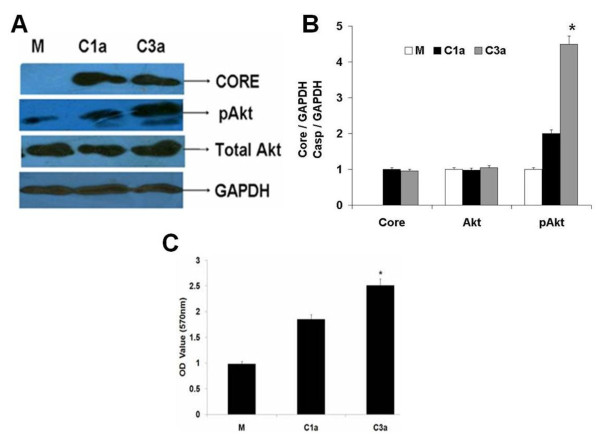
**HCV 3a Core gene increases p-Akt at protein level and increases cell proliferation**. **A) **The protein expression levels were determined by western blot analysis from Huh-7 cell lysates transfected with HCV Core vectors of genotype 1a, 3a and effect on p-Akt was analyzed using specific antibodies. GAPDH was used as internal control for normalization in western blotting. **B) **Blots were also normalized by measuring the amount of GAPDH (histogram). Bars are mean of optical density ratio of each group.* p < 0.001 verses control and Core 1a. GAPDH gene was used as internal control for normalization in western blotting. **C) **MTT assay showing the proliferation of Huh-7 cells transfected with Mock. HCV-core 3a and Core 1a, harvested at 48 hr and ob Otsuka M, served at 570 nm wavelength. All experiments were performed in 3 independent experiments having triplicate samples in each. Error bars indicate, mean S.D, *p < 0.01 verses mock.

It has been reported that activated Akt, a proximal downstream effecter of the anti-apoptotic phosphatidylinositol-3-kinase (PI3K) signaling pathway is critical in anti-apoptotic signaling [19,34]. In order to observe whether 1a and 3a differentially regulate the expression of phosphorylated-Akt (p-Akt), Huh-7 cells were transiently transfected with or without HCV genotype 1a and 3a Core expressing plasmids for 48 hrs. Significant increase in the protein expression levels in HCV 3a genotype was observed as compared to HCV 1a. Cellular extracts from Huh 7 cells were consecutively immuno detected using Core, Akt and p-Akt (Ser 473) specific antibodies. (Figure 3A). The results showed that total Akt protein levels were same for both genotypes while only the p-Akt was increased. These results suggested that HCV 3a Core protein activated Akt by phosphrylation of Akt which shows that HCV 3a core has stronger effect on activation of Akt as compared to HCV-1a Core in Huh-7 which is cell survival gene and enhanced cell growth. By densitometer we confirmed that there was 45% increase in protein level of p-Akt in Huh-7 cells transfected with Core 3a as compare to mock while expression of total Akt was same (Figure 3B).

It has been reported that HCV Core enhances proliferation with activation of p-Akt and increased levels of proinflammatory molecule PGE2 which in turn promote the growth of human HCC cells via VEGF [7]. So here we determined as an indirect effect of HCV Core genotype 1a and 3a on activation of Akt and cell proliferation through MTT cell proliferation assay. Results indicated significant enhanced cell proliferation with HCV 3a genotype Core as compared with HCV 1a genotype Core (Figure 3C).

## Discussion

Kinetics and extent of hepatocytes apoptosis as well as the pro- and anti-apoptotic mechanisms involved in HCV infection remain unclear. Different HCV genotypes and quasispecies may induce different effects, and studies on the contribution of genotypes or quasispecies to the effects on apoptosis are largely missing. Overall, the data regarding the role of different HCV proteins are controversial and attribute to a given viral protein pro- and anti-apoptotic effects, depending on the experimental system used. Several molecular interactions between Core protein and cellular genes of the apoptotic machinery have been found [13]. Different pro- and anti-apoptotic effects of the HCV Core protein from an individual patient have been described [33], suggesting special properties of different quasispecies proteins. Other studies showed Core-induced apoptosis through mitochondrial Cyto C release and indirect activation of bax TRAIL-induced apoptosis in hepatoma cells seems enhanced by Core-dependent bid-cleavage [24]. Taken together, it remains unclear whether HCV Core protein inhibits or induces death receptor-mediated apoptosis of hepatocytes. Our results are consistent to these all experiments in which HCV Core is found to inhibit apoptosis, our results showed that HCV Core 3a inhibits apoptosis by down regulating the expression of caspases (Caspase-3, 7, 8, 9 and 10) Cyto C and p53 which are involved in apoptotic pathways (Figure 1). Furthermore, on comparative analysis of gene expression by real time we observed significant reduction in HCV Core 3a transfected cells. Our results showed that HCV 3a Core down regulates the expression of these genes more than Core of HCV genotype 1a. As Caspase-3 is the most important down stream effecter triggered by Caspase-8, a vital component of the extrinsic cell death pathway, the down-regulation of Caspase-3 was further confirmed by observing the reduction of Caspase-3 at protein level by HCV 3a Core (Figure 2). Monitoring of Caspase activation might provide a reliable diagnostic tool to detect the degree of HCV-mediated inflammatory liver damage and to evaluate the efficacy of HCV therapy.

These finding are most important as our previous study of oxidative stress leading to HCC pathway has shown that HCV 3a Core has up-regulated the activation of Akt by its phosphorylation, also increasing PGE-2 production and cell proliferation as compared to HCV Core 1a [27]. We also confirmed that activation of Akt was more in HCV Core 3a transfected liver cells as compare to HCV Core 1a (Figure 3). Further we analyzed the effect of HCV Core 1a and 3a in Huh7 cells and found HCV Core 3a increased cell proliferation as compare to HCV Core 1a (Figure 3). Our results indicated that HCV Core protein promoted activated the anti-apoptotic signaling pathway including Akt and caspase pathway thus linking anti-apoptotic signaling with transcription machinery. This molecular mechanism of anti-apoptotic model by HCV Core protein might play a critical part of hepatocyte growth regulation and development of human HCC mediated by chronic HCV infection. Consistent with the "dual-signal" hypothesis postulating coupling of the proliferation pathways with those of cell death [35,36], these studies support our hypothesis that HCV Core protein triggers two mechanisms: an anti-apoptotic PI(3)K/Akt pathway and increases in cell proliferation. In fact, Bataller *et al*. recently demonstrated that expression of HCV Core protein in hepatic stellate cells increased cell proliferation in a Ras/ERK and PI(3)K/Akt-dependent manner. Recently, it has been reported that PI(3)K/Akt pathway was active in human liver tumors and in cultured hepatoma cells leading to proliferation of cells [37]. In agreement with these results, a number of previous investigations indicated that the inhibition of Akt in many hepatoma cell lines leads to growth arrest. Although the mechanisms explaining the activation of Akt in hepatoma cells are still unclear, we demonstrated that expression of HCV Core 3a protein resulted in much higher Akt activity and increase cell proliferation which may lead to HCC.

By taking together these all results it is confirmed that HCV Core 3a is mainly involved in decreased apoptosis and enhanced cells growth which can lead to HCC. As it has been shown that HCV genotype 3a is mainly associated with HCC in Pakistan [38] but mechanism was not understood. Previously, we reported that HCV genotype 3a causes oxidative stress more than genotype 1a and it is involved in HCV pathogenesis as HCV Core 3a increased the expression of genes which are involved in HCV pathogenesis indeed HCC [27]. HCV induced oxidative stress and steatosis is involved in DNA damage and HCC [39]. This study also confirms that HCV Core 3a is involved in HCV induced HCC by reduction of apoptosis and induction of cell proliferation by activation of Akt which is mainly involved in cell survival and cell growth. In conclusion, the present study for the first time suggest an interaction of HCV Core 3a for regulating the genes involved in HCV pathogenesis which may provide a better understanding for genome-specific mechanisms involved in disease progression by regulating caspase pathways, Tumor suppressor gene p53 and Phosphorylation of Akt which directly or indirectly increases cell proliferation leading to HCC.

## Methodology

### Source of samples

The local HCV 1a and 3a patient's serum samples used in this investigation were obtained from the CAMB (Center for Applied Molecular Biology) diagnostic laboratory, Lahore and Jinnah Hospital Lahore, Pakistan after quantification and genotype determination. Serum samples were stored at -80°C prior to RNA extraction for cloning and viral inoculation experiments. Patient's written consent and approval for this study was obtained from institutional ethics committee.

### Cell culture and transfection

Huh-7 cell line was kindly provided by Dr. Zafar Nawaz (University of Miami, USA) and maintained in Dulbecco's modified eagle medium (DMEM) supplemented with 100 μg/ml penicillin; streptomycin and 10% fetal bovine serum referred as complete medium (Sigma Aldrich, USA) at 37°C with 5% CO_2_. The medium was renewed every 3 day and passaged every 4-5 days. Briefly, cells were seeded in 24-well (1 × 10^5^/well) or 6-well (5 × 10^5^/well) plates and cultured in complete medium until they became 60-80% confluent. As We have described earlier that cells in 24-well plates were transiently transfected with 0.4 μg of HCV-1a and 3a constructed vectors [27,40], in serum free media using Lipofectamine™ 2000 (Invitrogen Life technologies, CA) according to the manufacturer's protocol. After 6hrs of incubation at 37°C in 5% CO_2_, complete medium was added to the cells. Protein analysis was carried out in 6-well plates. Cells were harvested at **48 hrs **post-transfection for gene expression analysis.

### Total RNA isolation and gene expression analysis

Total RNA from transfected and non-transfected cells was isolated using TRIzol reagent (Invitrogen life technologies, CA), 48 hrs post-transfection, cDNA was synthesized with 1 μg of total RNA using Superscript III cDNA synthesis kit (Invitrogen life technologies, CA) and semi-quantitative RT-PCR was done using specific primers described in (Table 1) for celluar genes involved in apoptosis and GAPDH as control. Quantitative Real Time PCR was carried out using Real Time ABI 7500 system (Applied Biosystems Inc, USA) with SYBR Green mix (Fermentas International Inc, Canada) (Table 1). The relative gene expression analysis was carried out by the SDS 3.1 software (Applied Biosystems Inc, USA). Each individual experiment was performed in triplicate.

**Table 1:** Sequences of primers used in Real Time PCR

No	Gene	Primer
**1**	Cap 3-F	ATGGAAGCGAATCAATGGAC
**2**	Cap3-R	GCCATGTCATCATCAACACC
**3**	Cap 8-F	TATGGCACTGATGGACAGGA
**4**	Cap8-R	GCAGAAAGTCAGCCTCATCC
**5**	Cap9-F	ATGTCGTCCAGGGTCTCAAC
**6**	Cap9-R	GGAAACTGTGAACGGCTCAT
**7**	Cap10-F	AGTGACAGGTATGGGCGTTC
**8**	Cap10-R	GCAGCACCTCAACTGTACCA
**9**	Cyt-F	ATTGGCGGCTGTGTAAGAGT
**10**	Cyt-R	CTGTCTACGGCACAGATGGA
**11**	p53-F	GGCCCACTTCACCGTACTAA
**12**	p53-R	GTGGTTTCAAGGCCAGATGT
**13**	GAPDH-F	ACCACAGTCCATGCCATCAC
**14**	GAPDH-R	TCCACCACCCTGTTGCTGTA

### Western blotting

To determine the effect of HCV Core gene on protein expression levels of caspase in the transfected and non-transfected cells, cells were lysed using ProteoJET mammalian cell lysis reagent (Fermentas, Canada). Equal amounts of total proteins were subjected to electrophoresis on 12% SDS-PAGE and electrophoretically transferred to a nitrocellulose membrane according to the manufacturer's protocol (Bio-Rad, CA). After blocking non-specific binding sites with 5% skimmed milk, blots were incubated with primary monoclonal antibodies specific to HCV Core, Caspase-3 and GAPDH genes (Santa Cruz Biotechnology Inc, USA) and secondary Horseradish peroxidase-conjugated anti-goat anti-mouse antibody (Sigma Aldrich, USA). The protein expressions were evaluated using chemiluminescence's detection kit (Sigma Aldrich, USA).

### Cell Proliferation Assay

Approximately 2 × 103 Huh-7 cells were seeded into 96-well tissue culture plates for 48 hrs prior to transfection. Huh-7 cells were transiently transfected with 0.1 μg/well of HCV Core 1a and 3a plasmids and a negative control in serum-free media using Lipofectamine™ 2000 (Invitrogen) according to the manufacturer's protocol. After 6 hrs incubation at 37°C in 5% CO2, CCM (complete culture media) was added to the cells. The MTT colorimetric assay was performed to detect cell proliferation after 48 hrs of incubation. The absorbance of resulting formazan crystals (solubilized with DMSO) was read at 590 nm on ELISA plate reader.

### Statistical analysis

All statistical analysis was done using SPSS software (version 16.0, SPSS Inc). Data are presented as mean ± SD. Numerical data were analyzed using student's t-test and ANOVA. P value < 0.05 was considered statistically significant.

## List of abbreviations

HCC: Hepatocellular carcinoma; HCV: Hepatitis C virus; PEG-INF-α: pegylated interferon alpha; Cyto C: Cytochrome C

## Competing interests

The authors declare that they have no competing interests.

## Authors' contributions

SJ conceive the idea and performed all the lab work. SK, BI, WA, UAA and MHS helped SJ in all practical lab work, literature review and data analysis. SK, UAA and SJ wrote, critically reviewed and finalized the manuscript. SH provided all facilitates to complete this work. All authors read and approved the final manuscript.

## Authors' information

Shah Jahan (PhD Molecular Biology), Saba Khaliq (PhD Molecular Biology), Usman Ali Ashfaq (PhD Molecular Biology), Muhammad Hassan Siddiqui (M.Phil Molecular Biology) and Bushra Ijaz and Waqar Ahmad are research officer at CEMB. Sajida Hassan (PhD Molecular Biology) is principle investigator at CEMB, University of the Punjab, Lahore.

## Supplementary Material

Additional file 1 **Sequence analysis of HCV Core 1a and 3a protein clones**. Click here for file

Additional file 2 **Time course of HCV Core gene of genotype 1a and 3a A**. Click here for file

Additional file 3 **Time course effect of HCV Core of genotype 1a and 3a on cellular genes involved in apoptosis**. Click here for file

## References

[B1] Hoofnagle JH Course and outcome of hepatitis C Hepatology 2002 36 S21 29 10.1053/jhep.2002.36227 12407573

[B2] Alter MJ Epidemiology of hepatitis C Hepatology 1997 26 62S 65S 10.1002/hep.510260711 9305666

[B3] Giannini C Brechot C Hepatitis C virus biology Cell Death Differ 2003 10 Suppl 1 S27 38 10.1038/sj.cdd.4401121 12655344

[B4] Raja NS Janjua KA Epidemiology of hepatitis C virus infection in Pakistan J Microbiol Immunol Infect 2008 41 4 8 18327420

[B5] Idrees M Rafique S Rehman I Akbar H Yousaf MZ Butt S Awan Z Manzoor S Akram M Aftab M Hepatitis C virus genotype 3a infection and hepatocellular carcinoma: Pakistan experience World J Gastroenterol 2009 15 5080 5085 10.3748/wjg.15.5080 PMC2768888 19860002

[B6] Choo QL Richman KH Han JH Berger K Lee C Dong C Gallegos C Coit D Medina-Selby R Barr PJ Genetic organization and diversity of the hepatitis C virus Proc Natl Acad Sci USA 1991 88 2451 2455 10.1073/pnas.88.6.2451 PMC51250 1848704

[B7] Lin C Lindenbach BD Pragai BM McCourt DW Rice CM Processing in the hepatitis C virus E2-NS2 region: identification of p7 and two distinct E2-specific products with different C termini J Virol 1994 68 5063 5073 10.1128/jvi.68.8.5063-5073.1994 PMC236449 7518529

[B8] Reed KE Rice CM Overview of hepatitis C virus genome structure, polyprotein processing, and protein properties Curr Top Microbiol Immunol 2000 242 55 84 10.1007/978-3-642-59605-6_4 10592656

[B9] Moriya K Nakagawa K Santa T Shintani Y Fujie H Miyoshi H Tsutsumi T Miyazawa T Ishibashi K Horie T Oxidative stress in the absence of inflammation in a mouse model for hepatitis C virus-associated hepatocarcinogenesis Cancer Res 2001 61 4365 4370 11389061

[B10] Moriya K Fujie H Shintani Y Yotsuyanagi H Tsutsumi T Ishibashi K Matsuura Y Kimura S Miyamura T Koike K The core protein of hepatitis C virus induces hepatocellular carcinoma in transgenic mice Nat Med 1998 4 1065 1067 10.1038/2053 9734402

[B11] Bantel H Lugering A Poremba C Lugering N Held J Domschke W Schulze-Osthoff K Caspase activation correlates with the degree of inflammatory liver injury in chronic hepatitis C virus infection Hepatology 2001 34 758 767 10.1053/jhep.2001.28229 11584373

[B12] Bantel H Ruck P Gregor M Schulze-Osthoff K Detection of elevated caspase activation and early apoptosis in liver diseases Eur J Cell Biol 2001 80 230 239 10.1078/0171-9335-00154 11322387

[B13] Bantel H Schulze-Osthoff K Apoptosis in hepatitis C virus infection Cell Death Differ 2003 10 Suppl 1 S48 58 10.1038/sj.cdd.4401119 12655346

[B14] Ray RB Meyer K Steele R Shrivastava A Aggarwal BB Ray R Inhibition of tumor necrosis factor (TNF-alpha)-mediated apoptosis by hepatitis C virus core protein J Biol Chem 1998 273 2256 2259 10.1074/jbc.273.4.2256 9442069

[B15] Ruggieri A Harada T Matsuura Y Miyamura T Sensitization to Fas-mediated apoptosis by hepatitis C virus core protein Virology 1997 229 68 76 10.1006/viro.1996.8420 9123879

[B16] Machida K Tsukiyama-Kohara K Seike E Tone S Shibasaki F Shimizu M Takahashi H Hayashi Y Funata N Taya C Inhibition of cytochrome c release in Fas-mediated signaling pathway in transgenic mice induced to express hepatitis C viral proteins J Biol Chem 2001 276 12140 12146 10.1074/jbc.M010137200 11278624

[B17] Kao CF Chen SY Chen JY Wu Lee YH Modulation of p53 transcription regulatory activity and post-translational modification by hepatitis C virus core protein Oncogene 2004 23 2472 2483 10.1038/sj.onc.1207368 14968111

[B18] Otsuka M Kato N Lan K Yoshida H Kato J Goto T Shiratori Y Omata M Hepatitis C virus core protein enhances p53 function through augmentation of DNA binding affinity and transcriptional ability J Biol Chem 2000 275 34122 34130 10.1074/jbc.M000578200 10924497

[B19] Fang L Li G Liu G Lee SW Aaronson SA p53 induction of heparin-binding EGF-like growth factor counteracts p53 growth suppression through activation of MAPK and PI3K/Akt signaling cascades Embo J 2001 20 1931 1939 10.1093/emboj/20.8.1931 PMC125417 11296226

[B20] Otsuka M Kato N Taniguchi H Yoshida H Goto T Shiratori Y Omata M Hepatitis C virus core protein inhibits apoptosis via enhanced Bcl-xL expression Virology 2002 296 84 93 10.1006/viro.2002.1371 12036320

[B21] Muzio M Chinnaiyan AM Kischkel FC O'Rourke K Shevchenko A Ni J Scaffidi C Bretz JD Zhang M Gentz R FLICE, a novel FADD-homologous ICE/CED-3-like protease, is recruited to the CD95 (Fas/APO-1) death--inducing signaling complex Cell 1996 85 817 827 10.1016/s0092-8674(00)81266-0 8681377

[B22] Varfolomeev EE Schuchmann M Luria V Chiannilkulchai N Beckmann JS Mett IL Rebrikov D Brodianski VM Kemper OC Kollet O Targeted disruption of the mouse Caspase 8 gene ablates cell death induction by the TNF receptors, Fas/Apo1, and DR3 and is lethal prenatally Immunity 1998 9 267 276 10.1016/s1074-7613(00)80609-3 9729047

[B23] Fuentes-Prior P Salvesen GS The protein structures that shape caspase activity, specificity, activation and inhibition Biochem J 2004 384 201 232 10.1042/BJ20041142 PMC1134104 15450003

[B24] Chou AH Tsai HF Wu YY Hu CY Hwang LH Hsu PI Hsu PN Hepatitis C virus core protein modulates TRAIL-mediated apoptosis by enhancing Bid cleavage and activation of mitochondria apoptosis signaling pathway J Immunol 2005 174 2160 2166 10.4049/jimmunol.174.4.2160 15699147

[B25] Calabrese F Pontisso P Pettenazzo E Benvegnu L Vario A Chemello L Alberti A Valente M Liver cell apoptosis in chronic hepatitis C correlates with histological but not biochemical activity or serum HCV-RNA levels Hepatology 2000 31 1153 1159 10.1053/he.2000.7123 10796892

[B26] Fischer R Baumert T Blum HE Hepatitis C virus infection and apoptosis World J Gastroenterol 2007 13 4865 4872 10.3748/wjg.v13.i36.4865 PMC4611765 17828818

[B27] Jahan S Khaliq S Ijaz B Ahmad W Hassan S Role of HCV Core gene of genotype 1a and 3a and host gene Cox-2 in HCV-induced pathogenesis Virol J 8 155 10.1186/1743-422X-8-155 PMC3080829 21457561

[B28] Niwa H Yamamura K Miyazaki J Efficient selection for high-expression transfectants with a novel eukaryotic vector Gene 1991 108 193 199 10.1016/0378-1119(91)90434-d 1660837

[B29] Nishizono A Hiraga M Mifune K Terao H Fujioka T Nasu M Goto T Misumi J Moriyama M Arakawa Y Correlation of serum antibody titers against hepatitis C virus core protein with clinical features by western blot (immunoblot) analysis using a recombinant vaccinia virus expression system J Clin Microbiol 1993 31 1173 1178 10.1128/jcm.31.5.1173-1178.1993 PMC262898 7684748

[B30] Downward J PI 3-kinase, Akt and cell survival Semin Cell Dev Biol 2004 15 177 182 10.1016/j.semcdb.2004.01.002 15209377

[B31] Bartenschlager R Hepatitis C virus molecular clones: from cDNA to infectious virus particles in cell culture Curr Opin Microbiol 2006 9 416 422 10.1016/j.mib.2006.06.012 16814596

[B32] Dustin LB Rice CM Flying under the radar: the immunobiology of hepatitis C Annu Rev Immunol 2007 25 71 99 10.1146/annurev.immunol.25.022106.141602 17067278

[B33] Pavio N Lai MM The hepatitis C virus persistence: how to evade the immune system? J Biosci 2003 28 287 304 10.1007/BF02970148 12734407

[B34] Reynolds CM Eguchi S Frank GD Motley ED Signaling mechanisms of heparin-binding epidermal growth factor-like growth factor in vascular smooth muscle cells Hypertension 2002 39 525 529 10.1161/hy0202.103076 11882602

[B35] Harrington EA Bruce JL Harlow E Dyson N pRB plays an essential role in cell cycle arrest induced by DNA damage Proc Natl Acad Sci USA 1998 95 11945 11950 10.1073/pnas.95.20.11945 PMC21745 9751770

[B36] Evan GI Brown L Whyte M Harrington E Apoptosis and the cell cycle Curr Opin Cell Biol 1995 7 825 834 10.1016/0955-0674(95)80066-2 8608013

[B37] Wang GL Iakova P Wilde M Awad S Timchenko NA Liver tumors escape negative control of proliferation via PI3K/Akt-mediated block of C/EBP alpha growth inhibitory activity Genes Dev 2004 18 912 925 10.1101/gad.1183304 PMC395850 15107404

[B38] Idrees M Lal A Naseem M Khalid M High prevalence of hepatitis C virus infection in the largest province of Pakistan J Dig Dis 2008 9 95 103 10.1111/j.1751-2980.2008.00329.x 18419643

[B39] Moriya K Fujie H Yotsuyanagi H Shintani Y Tsutsumi T Matsuura Y Miyamura T Kimura S Koike K Subcellular localization of hepatitis C virus structural proteins in the liver of transgenic mice Jpn J Med Sci Biol 1997 50 169 177 10.7883/yoken1952.50.169 9556757

[B40] Khaliq S Jahan S Ijaz B Ahmad W Asad S Pervaiz A Samreen B Khan M Hassan S Inhibition of core gene of HCV 3a genotype using synthetic and vector derived siRNAs Virol J 7 318 10.1186/1743-422X-7-318 PMC2992066 21073745

