# Supplementary material for: Anti-apoptotic effect of HCV core gene of genotype 3a in Huh-7 cell line
Source: Virol J. 2011 Nov 23;8:522. doi: 10.1186/1743-422X-8-522 (PMC3247135; doi:10.1186/1743-422X-8-522)
Supplement: Additional file 1 — Sequence analysis of HCV Core 1a and 3a protein clones. [file 1743-422X-8-522-S1.DOC]

1. **Amino acid sequence comparison of HCV Core 3a and 1a**

After analyzing the effect of HCV core 1a and 3a on celluar genesat mRNA and protein level, we found that HCV core 3a has induced these cellular gens more than HCV core 1a. On the basis of these fonding we made hypothsis that variation of amino acid sequence between HCV core 1a and 3amay be responsible for variablae effect of both genotype .The amino acid sequence comparison showed that 3a Core protein has 90% similarity to 1a Core protein and 98% similarity to their consensus sequences. Beside reported amino acid substitutions, we found additional differences in both genotypes sequence analysis of HCV Core 1a and 3a protein clones. Comparison of full predicted amino acid sequence of HCV Core isolates in our study against a consensus genotype 3a clone (Con 3a) and 1a (Con 1a) from the HCV sequence database was performed using the ClustalW program **(Figure S1)**.

**(Figure S1 )**.


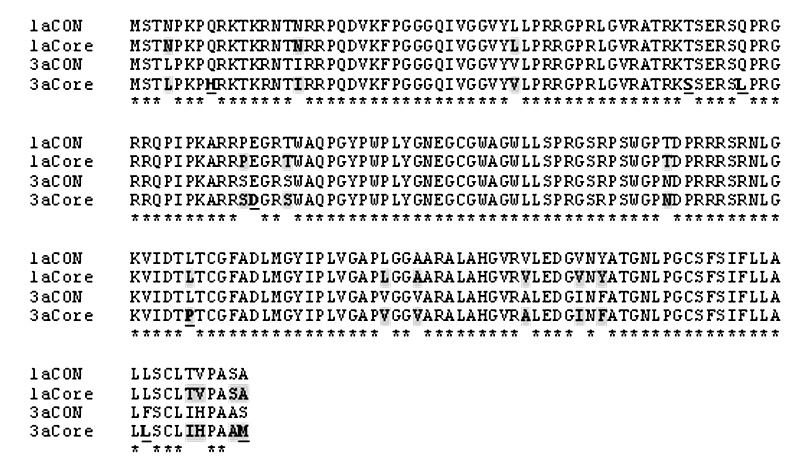


**Figure S1:**  **Sequence analysis of HCV Core 1a and 3a protein clones.** Comparison of full predicted amino acid sequence of HCV Core isolates in our study against a consensus genotype 3a clone (Con 3a) and 1a (Con 1a) from the HCV sequence database, performed using the ClustalW program. Sequences different from consensus are boldface and underlined. Differences in the individual amino acid in Core 1a and 3a are highlighted. Asterisks at the bottom of a sequence comparison indicate complete identity at that position.
