# Supplementary material for: Anti-apoptotic effect of HCV core gene of genotype 3a in Huh-7 cell line
Source: Virol J. 2011 Nov 23;8:522. doi: 10.1186/1743-422X-8-522 (PMC3247135; doi:10.1186/1743-422X-8-522)
Supplement: Additional file 2 — Time course of HCV Core gene of genotype 1a and 3a A. [file 1743-422X-8-522-S2.DOC]

1. **Time course expression analysis of HCV Core in Huh-7 cells**

At post-transfection with different time intervals of 24, 48, 72 and 96 hrs expression of pCR3.1 FlagTAG/Core (0.4µg/well) of genotypes 1a and 3a in Huh-7cells was relatively quantified by real time PCR. These results indicate that the maximum expression levels were achieved for HCV Core gene of genotype (1a and 3a) at 48hrs post-transfection that were 4.6 and 4 fold for the Core gene of genotype 3a and 1a respectively **(Figure S2A).** Similarly, highest level of expression (3 and 3.6 fold) were observed for E2 of genotype 3a and 1a as compare to mock using Real Time PCR **(Figure S2B)**.

**A**

**B**


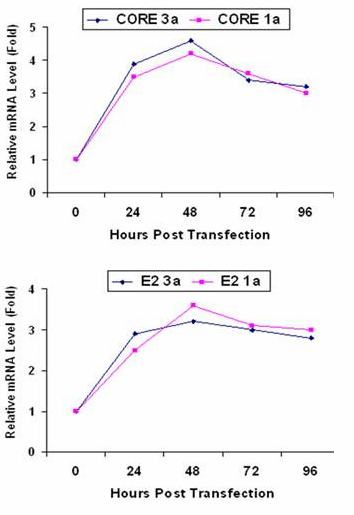


**Figure S2**: **Time course of HCV Core gene of genotype 1a and 3a A)** Expression of Core gene of genotype 1a and 3a in Huh-7 cells transfected with Core clone (0.4µg/well) at different time (24hr, 48hr, 72hr and 96hr) Cells were harvested and relative RNA determinations were carried out using semi-quantitative RT-PCR. **B)** Relative Gene Expression of Core gene of genotype 1a and 3a in Huh-7 cells transfected with Core clone (0.4µg/well) at different time (24hr, 48hr, 72hr and 96hr). Total cellular RNA extracted, was quantified by Real-Time PCR using gene specific primers in comparison to Mock. GAPDH was used as internal control.
