# Supplementary material for: Anti-apoptotic effect of HCV core gene of genotype 3a in Huh-7 cell line
Source: Virol J. 2011 Nov 23;8:522. doi: 10.1186/1743-422X-8-522 (PMC3247135; doi:10.1186/1743-422X-8-522)
Supplement: Additional file 3 — Time course effect of HCV Core of genotype 1a and 3a on cellular genes involved in apoptosis. [file 1743-422X-8-522-S3.DOC]

1. **Time course effect of HCV Core of genotype 1a and 3a on cellular genes involved in apoptosis**

Effect of HCV core of genotype 1a and 3a on celluar genes Casp 3, 8,9,10 and Cyto-C at different time intervals of 24, 48, 72 and 96 hrs post transfection were analyzed in Huh-7cells. Gene expression was relatively quantified by real time PCR. These results indicate that the maximum inhibition of cellular genes were achieved with HCV Core gene of genotype (1a and 3a) at 48hrs and 96 hr post-transfection that were almost similar Reduction in the expression of Caspase-3, 8, 9 and 10, and Cyto C genes were significant in HCV core 3a trasfected cell at 48 hr and 96 hr as compare to mock transfected cells while expression of cellular gene were almost same at 48 hr and 96. **(Figure S3A).** There was no significant effect of HCV core of genotype 1a on cellular genes involved in Apoptosis **(Figure S3B)**.

**(Figure S3A)**.


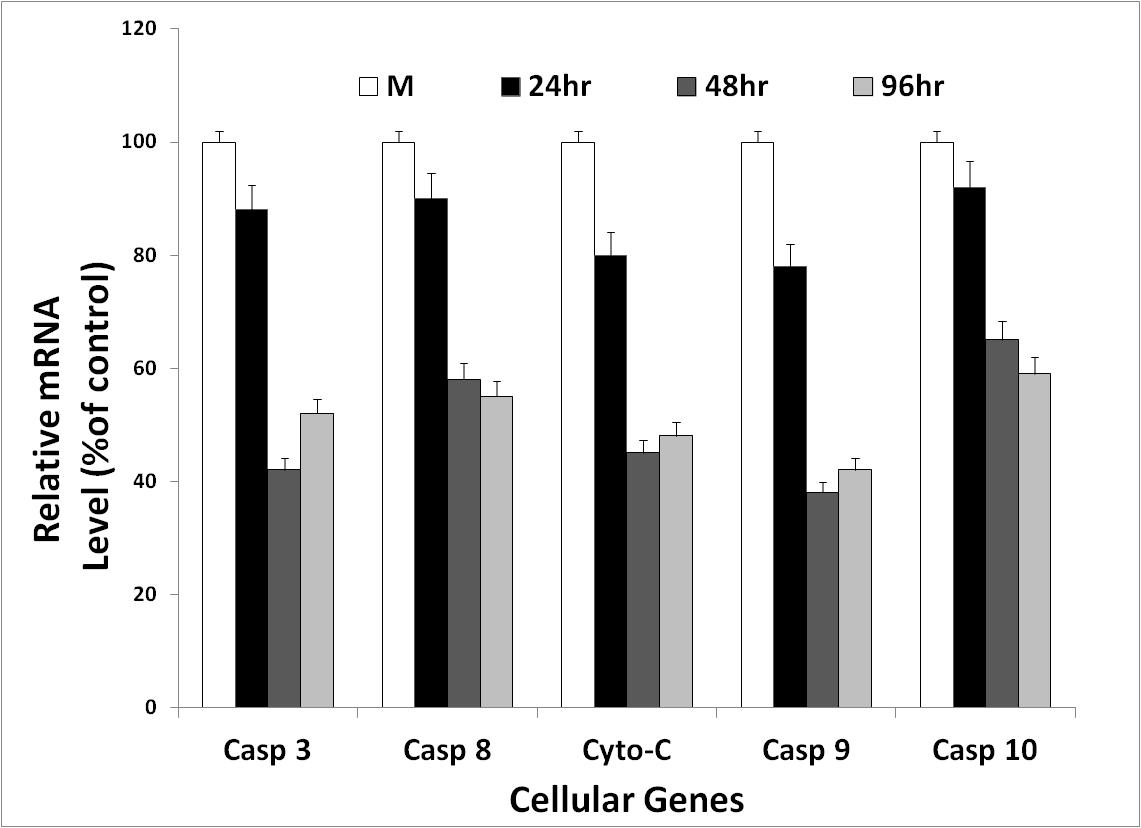


**(A)**Comparison of gene expression Caspase-3, 8, 9, 10, and Cyto C genes of in transiently transfected Huh-7 cells with HCV core of genotype 3a at 24, 48 and 96 hr post transfection. All experiments were performed in 3 independent experiments having triplicate samples in each. Error bars indicate, mean S.D, *p<0.01 verses mock

**(Figure S3B)**.


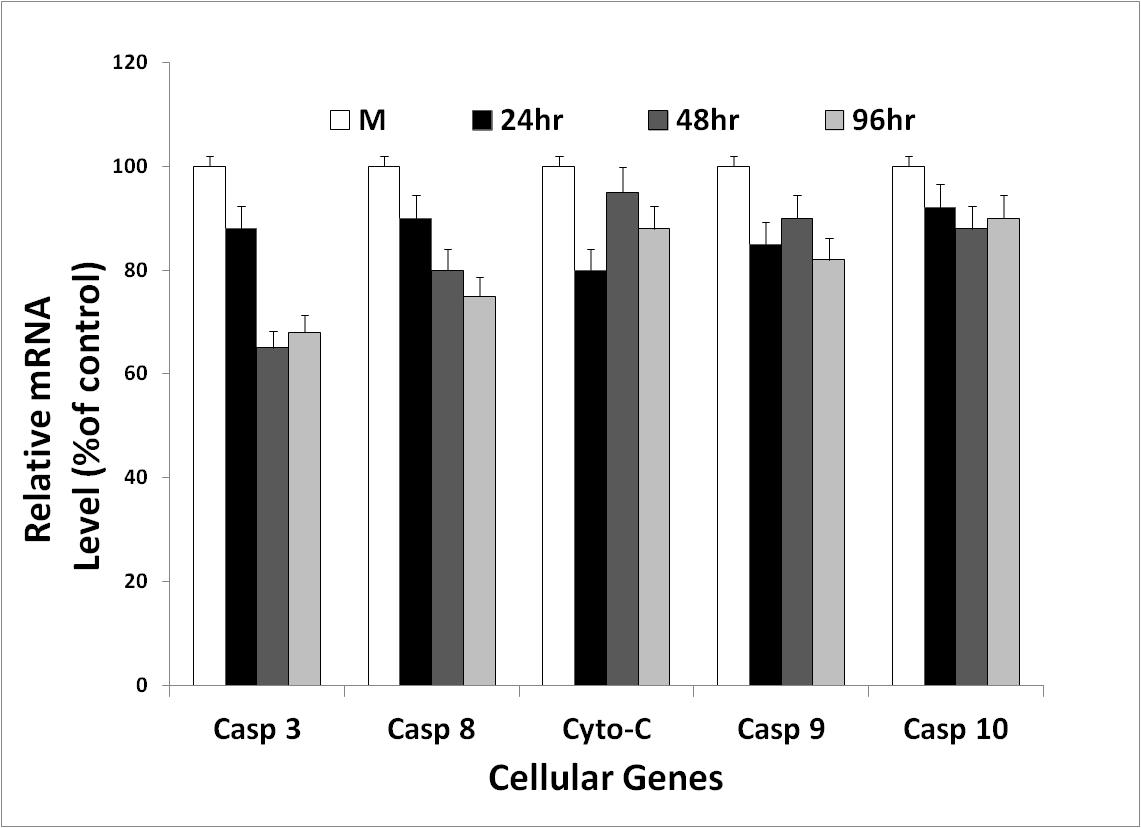


**(B)** Comparison of gene expression Caspase-3, 8, 9, 10, and Cyto C genes of in transiently transfected Huh-7 cells with HCV core of genotype 1a at 24, 48 and 96 hr post transfection. All experiments were performed in 3 independent experiments having triplicate samples in each. Error bars indicate, mean S.D, *p<0.01 verses mock.
